# Vascular diameter and intima-media thickness to diameter ratio values of the carotid artery in 642 healthy children

**DOI:** 10.1007/s00431-020-03785-3

**Published:** 2020-09-18

**Authors:** Luisa Semmler, Heidi Weberruß, Lisa Baumgartner, Raphael Pirzer, Renate Oberhoffer-Fritz

**Affiliations:** 1 Institute of Preventive Pediatrics, Technical University, Munich, Germany; 2 Department of Anaesthesiology and Operative Intensive Care, University Hospital, Augsburg, Germany

**Keywords:** Cardiovascular disease, Carotid intima-media thickness/diameter ratio, Children, Vascular diameter

## Abstract

In children, arterial alterations occur with increased intima-media thickness as well as vascular diameter enlargement. Both conditions correlate with higher cardiovascular risk in adults, and both the array and proportion of these alterations are important hemodynamic parameters. In terms of functional adaptation processes, they influence several arterial wall properties as for example the shear and tensile stress of the vessel. There are no reference values for the vascular diameter and intima-media thickness/diameter ratio of the carotid artery in children. Therefore, this study aimed to assess vascular diameter, intima-media thickness/diameter ratio and related tensile stress values in children and to further investigate the influence of sex, age, body mass index, and blood pressure. The parameters were measured with high-resolution semi-automated ultrasound. Sex- and age-dependent values were calculated with the LMS method for a cross-sectional sample of 642 healthy, non-obese children aged 8–17 years. The mean vascular diameter was 5.45 ± 0.46 mm; the median intima-media thickness/diameter ratio was 0.085 (0.079–0.092); the mean tensile stress was 66.0 ± 8.8 kPa. The vascular diameter and the tensile stress were higher, and the intima-media thickness/diameter ratio was lower in boys than in girls. In comparison to the normal weight study population the excluded obese children had a significantly higher diameter, a lower intima-media thickness/diameter ratio, and a higher tensile stress. In multiple regression analyses of diameter, intima-media thickness/diameter ratio, and tensile stress, all parameters were influenced by sex and body mass index. Furthermore, systolic and diastolic blood pressure significantly influenced the vascular diameter, and systolic blood pressure significantly influenced the intima-media thickness/diameter ratio.

*Conclusion*: This study is the first to report values for the diameter, the intima-media thickness/diameter ratio of the carotid artery, and the related tensile stress allowing a more differentiated view of cardiovascular adaptations as it combines structural and functional vascular parameters.

**Table:** 

What is known: *• Intima-media thickness and vascular diameter are related to a higher cardiovascular risk in adults* *• The intima-media thickness/diameter ratio gives information about hemodynamic and functional vessel adaptation* What is new: *• Values for vascular diameter, intima-media thickness/diameter ratio, and tensile stress of the carotid artery in children are presented in this study* *• Intima-media thickness as a surrogate marker for arterial health in children should be complemented by intima-media thickness/diameter ratio measurement*

**Electronic supplementary material:**

The online version of this article (10.1007/s00431-020-03785-3) contains supplementary material, which is available to authorized users.

## Introduction

Cardiovascular disease (CVD) accounts for approximately one-third of all deaths globally [1]. The leading cause of CVD is atherosclerosis, which may begin in childhood [2]. Carotid intima-media thickness (IMT) is an established surrogate marker to detect an altered arterial wall structure and subclinical atherosclerosis in children and adults [3]. Known risk factors for CVD are obesity [4], hypertension [5], familial hypercholesterolemia [6], type 1 diabetes [7], non-alcoholic fatty liver disease [8], chronic kidney disease [9], and inflammatory factors, such as HIV infection, treated with antiretroviral therapy [10], and increased serum C-reactive protein concentrations [11]. All these risk factors are associated with increased IMT in children. Moderate physical activity, a protective factor for CVD [12], was shown to decrease IMT in children [13]. In contrast, exercise can also lead to an increased IMT [14, 15] and was positively associated with cardiorespiratory fitness in children [16, 17]. Besides IMT, further vascular parameters exist that describe arterial properties. For example, in adults, there is an independent association between increased vascular diameter (D) and higher cardiovascular risk [18, 19]. Respectively, a larger D of the common carotid artery (CCA) in childhood is associated with chronic kidney disease [9], obesity [20], metabolic syndrome [21], and hypertension [22]. The ratio between IMT and D can be specified as the intima-media thickness/diameter ratio (IDR). Adult arteries may adapt by increasing D to compensate for an atherosclerotic-induced thickening of IMT; thus, hemodynamically significant vessel narrowing can be prevented [23]. In a hemodynamic approach, shear and tensile stress are relevant determinants for arterial alterations. Shear stress refers to the velocity near the arterial wall that aligns the endothelium with the flow direction [24], and is influenced by viscosity, blood flow, and D [25]. Tensile stress is regarded as a stretching force perpendicular to a longitudinal section of the arterial wall and is approximated by distending pressure, D, and IMT [26]. Vessels can adapt by increasing D to restore altered shear stress induced for example by a higher flow rate. IMT may subsequently increase to maintain tensile stress [24, 27]. Because adaptations of IMT and D may not always occur simultaneously, monitoring both parameters and measuring their ratio, defined as IDR, can offer a more differentiated view of vascular changes. These parameters may indicate whether IMT and D adapt in terms of adequate remodeling or within a pathologic process [28, 29]. In contrast to IMT, for which reference values exist [30–32], there are no reference values for D and IDR in children. Therefore, this study aimed to calculate sex- and age-dependent values for D, IDR, and related tensile stress of the CCA for 642 healthy children. Furthermore, the influence of sex, age, body mass index (BMI), and systolic and diastolic blood pressure (SBP, DBP) on D, IDR, and tensile stress was analyzed.

## Materials and methods

### Participants

The study was approved by the local ethics committee (5490/12) and met the ethical guidelines of the Declaration of Helsinki (revision 2013). Written informed consent was obtained from all children aged ≥ 14 years and all participants’ parents.

### Anthropometry and blood pressure

Measurements of body mass and height were made to the nearest 0.1 kg and 0.1 cm (seca 799; seca, Hamburg, Germany), respectively, both without shoes and wearing light clothes. The BMI was calculated by body mass (kg) / height (m)^2^. Children with a BMI ≥ 90th percentile were considered overweight, and those with a BMI ≥ 97th percentile as obese [33]. After 10 min of rest, peripheral SBP and DBP were obtained using an oscillometer (Mobil-O-Graph, I.E.M.) on the left upper arm. All measurements were conducted by trained staff. Children with a single measurement > 95th percentile [34] were not diagnosed with manifest, but with suspected hypertension. Mean arterial pressure (MAP) was calculated with the following formula: MAP = (DBP × 2 + SBP) / 3 [35].

### IMT, D, and tensile stress

IMT measurements were recorded using high-resolution, non-invasive, semi-automated B-Mode ultrasound. D was measured with the same device in M-Mode (ProSound Alpha 6; Aloka/Hitachi Medical Systems), both with a high-frequency linear array probe (5–13 MHz). The IMT was measured on the far wall of the CCA, 1 cm proximal to the bulb at the end-diastolic moment (R-wave), when IMT is thickest [36]. The vascular diameter was measured at the same location as IMT. The average minimum D value of the left and right CCA, which corresponds with end-diastolic IMT, was recorded from at least five heart cycles [36]. The IDR was calculated as the ratio between IMT and D. Further details of the measurement methodology are described elsewhere [31]. Tensile stress was calculated as MAP × D / 2 × IMT [37].

### Statistical analysis

Statistical analysis was performed with the statistical software R Studio (version 1.1. 463, 2009-2018, RStudio). The parameters of the study population were expressed as mean ± standard deviation (SD) or median and interquartile range (IQR) according to their distribution. The participants were clustered into five age groups (7.75–10.00, 10.00–11.99, 12.00–13.99, 14.00–15.99, and 16.00–17.25 years). Sex differences in anthropometric data and sex-dependent differences concerning D, IDR, and tensile stress were analyzed using an independent two-sample *t* test or Mann-Whitney *U* test. Participants were furthermore clustered into three BMI groups, separated by sex (normal weight: BMI < 90th percentile, overweight: BMI ≥ 90th and < 97th percentile, obese: BMI ≥ 97th percentile). The z-scores of D, IDR, and tensile stress were calculated for each BMI level (normal weight, overweight, and obese) according to the following formula:

$$\begin{array}{l}\mathrm z-\mathrm{Score}=\dfrac{\left[\left(\frac xM\right)^{L}\right]{-1}}{L\times S}\;\mathrm{for}\;L\neq0\;\mathrm{or}\\\mathrm z-\mathrm{score}=\dfrac{\ln\left(\frac xM\right)}S\;\mathrm{for}\;L=0\end{array}$$

The differences in z-scores regarding D, IDR, and tensile stress were calculated by an one-way analysis of variance or a Kruskal-Wallis test. The influence of sex, age, BMI, SBP, and DBP on D, IDR, and tensile stress was analyzed by a multiple linear regression. Sex- and age-dependent values for D, IDR, and tensile stress were estimated using GAMLSS (Generalized Additive Models for Location, Scale, and Shape) software, applying the LMS method [38]. A Box-Cox Cole and Green distribution was assumed for the response variable to transform data into a normal distribution. The approximate median (M), the approximate coefficient of variation (S), and skewness (L) were all estimated [39]. A *p* value < 0.05 was considered statistically significant.

## Results

### Participants

**Fig. 1:**
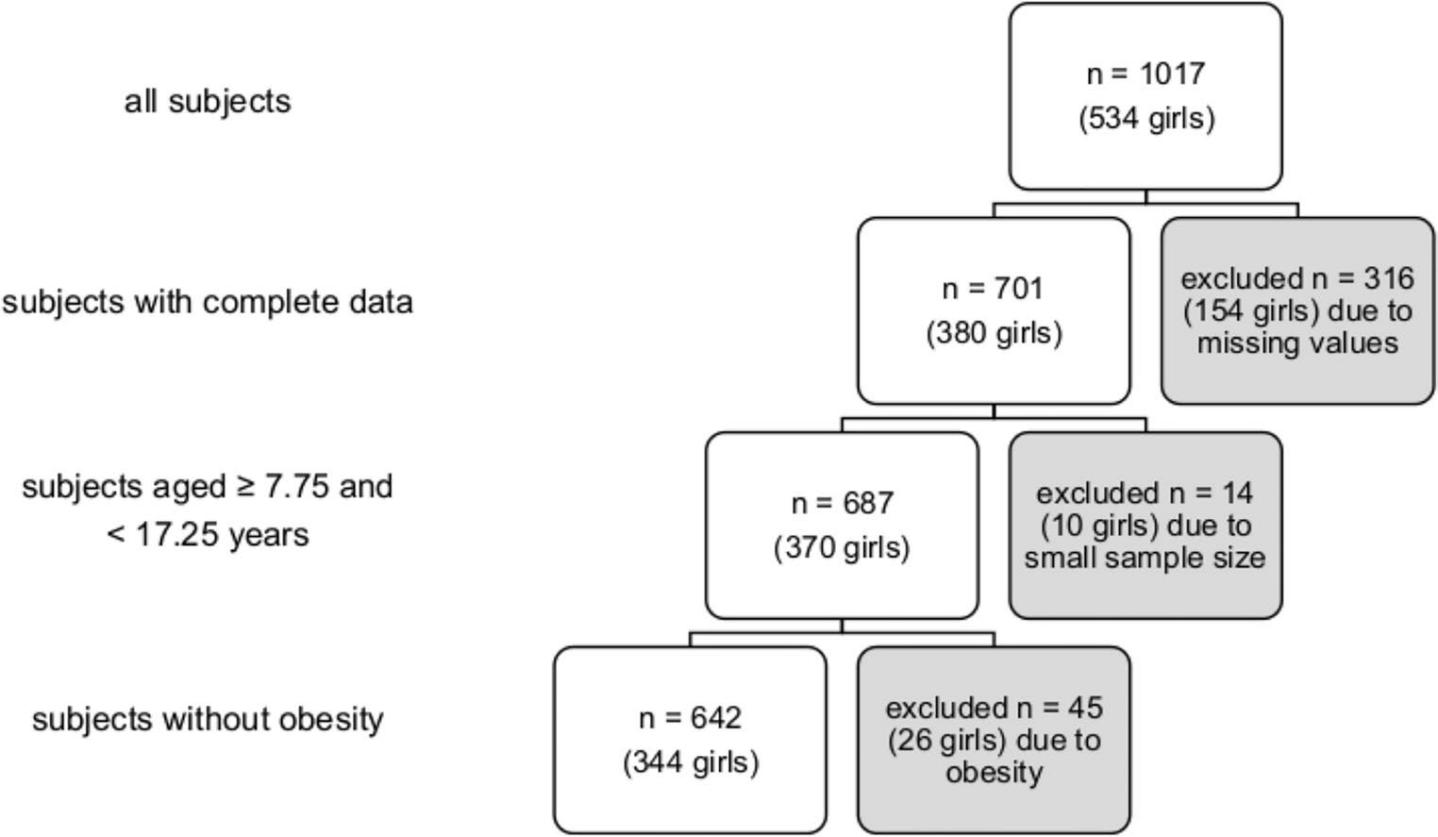
Inclusion and exclusion of participants

Data were collected from October 2012 to July 2013 as part of the project “Sternstunden der Gesundheit” in a school-based setting in the region Berchtesgadener Land, Germany [31]. In total, 1017 healthy schoolchildren aged 7–18 years were examined. Due to technical problems, data were lost on IMT for 264 children, on D for 89 children, and on BP for one child. Furthermore, 14 participants aged < 7.75 years or ≥ 17.25 years were excluded due to inadequate sample sizes. In addition, 45 obese children (BMI > 97th percentile [33]) were excluded from the analysis but considered for the comparison of D, IDR, and tensile stress between the different BMI groups. All together, valid data were available for 642 non-obese participants age 7.75–17.25 years (Fig. 1). For IMT, intertester variability was 4.79%.

Female participants were significantly older (*p* < 0.001) and taller (*p* = 0.006) than male participants. For more details on the study population’s characteristics, see Table 1. Nineteen girls and 30 boys were overweight, and 26 girls and 19 boys obese.

**Table 1:** Characteristics of study participants

	Total	Girls	Boys	
	*n* = 642	*n* = 344	*n* = 298	
	Mean ± SD or median (IQR)	Mean ± SD or median (IQR)	Mean ± SD or median (IQR)	*p* value
Age (year)	11.9 (10.6–13.9)	12.3 (10.6–14.3)	11.6 (10.5–12.8)	< 0.001
Height (cm)	153.0 (142.0–163.0)	156.0 (143.5–164.0)	150.5 (140.5–160.0)	0.006
Body mass (kg)	42.6 (33.4–51.9)	44.3 (33.8–52.4)	40.3 (33.0–50.1)	0.051
BMI (kg/m^2^)	17.9 (16.3–20.2)	18.2 (16.4–20.1)	17.8 (16.2–20.3)	0.520
SBP (mmHg)	115.0 (109.3–122.0)	115.0 (109.0–122.0)	115.0 (110.0–121.0)	0.872
DBP (mmHg)	67.9 ± 8.1	68.1 ± 8.2	67.6 ± 8.0	0.440
MAP (mmHg)	83.8 ± 7.6	84.0 ± 7.8	83.6 ± 7.5	0.576
IMT (mm)	0.46 ± 0.03	0.46 ± 0.03	0.46 ± 0.03	0.559
D (mm)	5.45 ± 0.46	5.35 ± 0.42	5.58 ± 0.47	< 0.001
IDR	0.085 (0.079–0.092)	0.087 (0.081–0.093)	0.083 (0.078-0.090)	< 0.001
Tensile stress (kPa)	66.0 ± 8.8	64.9 ± 8.4	67.2 ± 9.1	< 0.001

Descriptive statistic of the study population with p-values indicating sex differences

*BMI* body mass index, *D* vascular diameter, *DBP* diastolic blood pressure, *IDR* intima-media thickness/diameter ratio, *IMT* intima-media thickness, *IQR* interquartile range, *MAP* mean arterial pressure, *SBP* systolic blood pressure, *SD* standard deviation

### Diameter

The mean D was 5.45 ± 0.46 mm for the total study population, 5.35 ± 0.42 mm for girls, and 5.58 ± 0.47 mm for boys. Mean ± SD or median (IQR) values of D for the particular age groups are shown in Table S1 (Online Resource). Boys had significantly higher D values than girls in total and in all age groups except 14.00–15.99 years (for all *p* < 0.05).

**Fig. 2:**
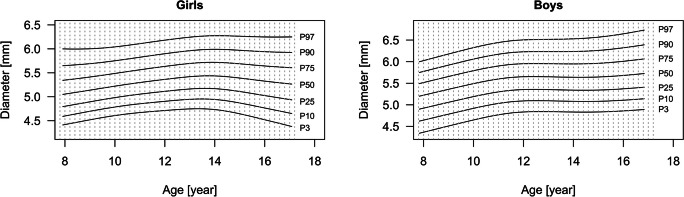
Smoothed percentiles of vascular diameter (D) for girls and boys aged 8–17 years

Age-dependent values and corresponding L, M, and S scores for D are specified in Table S2 for girls (Online Resource) and Table S3 for boys (Online Resource). Corresponding smoothed fitted percentiles are presented in Fig. 2.

In multiple regression analysis, female sex and SBP were negatively associated (*β* = − 0.26, *p* < 0.001; *β* = − 0.12, *p* = 0.006, respectively), and BMI and DBP were positively associated with D (*β* = 0.39, *p* < 0.001; *β* = 0.11, *p* = 0.010, respectively). Age was not significantly associated with D (Table 2).

**Table 2:** Multiple linear regression model for vascular diameter (D) and intima-media thickness/diameter ratio (IDR) and tensile stress.

	*β*	*β* st	*p* value	95% CI
Model 1: D, *R*^2^ = 0.22, p < 0.001				
Age	0.00	0.00	0.962	− 0.02 to 0.02
Female sex	− 0.24	− 0.26	< 0.001	− 0.30 to − 0.17
BMI	0.07	0.39	< 0.001	0.05 to 0.08
SBP	− 0.01	− 0.12	0.006	− 0.01 to 0.00
DBP	0.01	0.11	0.010	0.00 to 0.01
Model 2: IDR, *R*^2^ = 0.09*, p* < 0.001				
Age	0.00	0.06	0.204	0.00 to 0.00
Female sex	0.00	0.18	< 0.001	0.00 to 0.00
BMI	0.00	− 0.24	< 0.001	0.00 to 0.00
SBP	0.00	0.17	< 0.001	0.00 to 0.00
DBP	0.00	− 0.08	0.097	0.00 to 0.00
Model 3: tensile stress, *R*^2^ = 0.06*, p* < 0.001				
Age	0.04	0.01	0.809	− 0.30 to 0.39
Female sex	− 2.38	− 0.13	< 0.001	− 3.73 to − 1.03
BMI	0.68	0.21	< 0.001	0.40 to 0.97

Multiple regression analysis of D, IDR**,** and tensile stress with different influencing, anthropometric variables. Model 1: dependent variable: D, independent variables: age, female sex, BMI, SBP, DBP; Model 2: dependent variable: IDR, independent variables: age, female sex, BMI, SBP, DBP; Model 3: dependent variable: tensile stress, independent variables: age, female sex, BMI

*BMI* body mass index, *CI* confidence interval, *D* vascular diameter, *DBP* diastolic blood pressure, *IDR* intima-media thickness/diameter ratio, *SBP* systolic blood pressure, *β* regression coefficients, *β st* standardized regression coefficients, *R*^2^ adjusted R^2^

**Fig. 3:**
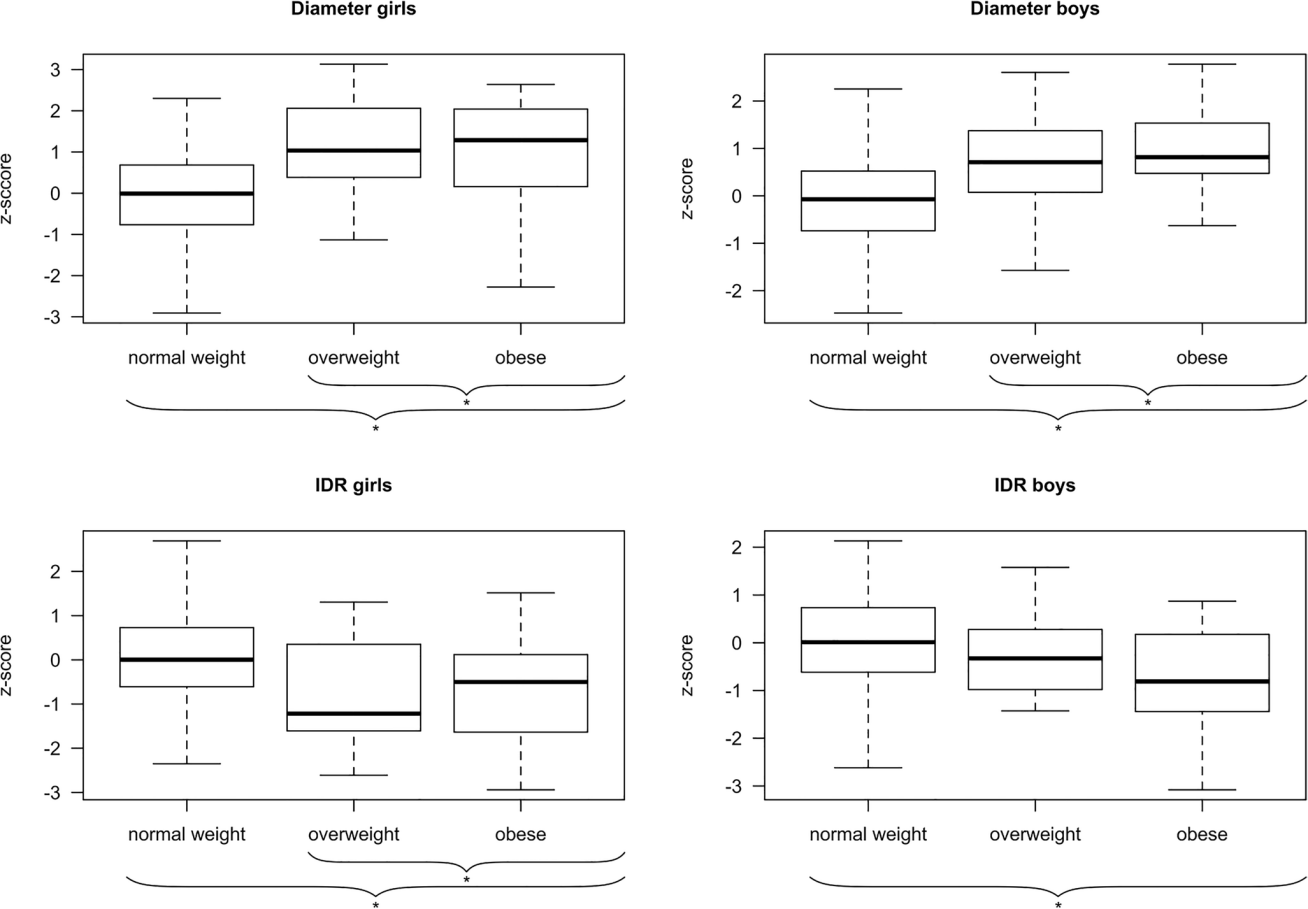
Z-scores for vascular diameter (D) and intima-media thickness/diameter ratio (IDR) in normal weight, overweight, and obese girls and boys; *p < 0.05

Comparing the z-scores of D between the BMI groups, overweight and obese participants showed significantly higher z-scores for D versus normal weight participants (for all *p* < 0.001: Fig. 3, Table S4 Online Resource).

### IMT and IDR

The IMT, but not the IDR, was normally distributed in the study population. The mean IMT of the total study population, and in both girls and boys, was 0.46 ± 0.03 mm. The median IDR was 0.085 (0.079–0.092) for the entire study population, and 0.087 (0.081-0.093) for girls and 0.083 (0.078-0.090) for boys, respectively. Mean ± SD or median (IQR) values of IDR for the particular age groups are shown in Table S1 (Online Resource). Girls had a higher IDR than boys in total and in the age groups 10.00–11.99 years and 12.00–13.99 years (for all *p* < 0.05).

**Fig. 4:**
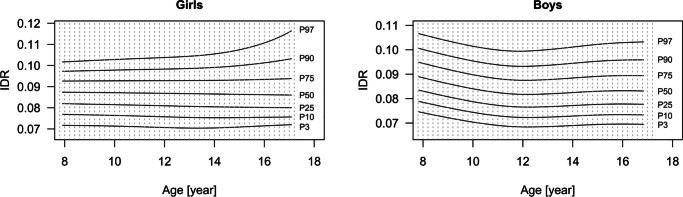
Smoothed percentiles of intima-media thickness/diameter ratio (IDR) for girls and boys aged 8–17 years

Age-dependent values and corresponding L, M, and S scores for IDR are specified in Table S5 (Online Resource) for girls and Table S6 for boys (Online Resource). Figure 4 presents the corresponding smoothed fitted percentiles.

In multiple regression analysis, female sex and SBP were positively associated with IDR (*β* = 0.18, *p* < 0.001; *β* = 0.17, *p* <0.001, respectively), whereas BMI was negatively associated with IDR (*β* = − 0.24, *p* < 0.001). Age and DBP did not have a significant influence on IDR (Table 2).

Comparing the z-scores of IDR between the BMI groups, in girls, overweight and obese participants showed significantly lower IDR z-scores versus normal weight children (for all *p* < 0.01: Fig. 3, Table S4 Online Resource). In boys, only obese children showed significantly lower IDR z-scores than normal-weight boys (*p* = 0.002).

### Tensile stress

The tensile stress was 66.0 ± 8.8 kPa for the total study population, 64.9 ± 8.4 kPa for girls and 67.2 ± 9.1 kPa for boys. Mean ± SD or median (IQR) values of tensile stress for the particular age groups are shown in Table S1 (Online Resource). Girls had significantly lower tensile stress values than boys in total and in the age groups of 10.00–11.99 years and 12.00-13.99 years (all *p* < 0.05). Age-dependent values and corresponding L, M, and S scores for tensile stress are specified in Table S7 for girls (Online Resource) and Table S8 for boys (Online Resource). Corresponding smoothed fitted percentiles are presented in Fig. S1 (Online Resource). In multiple regression analysis (Table 2), female sex was negatively associated with tensile stress (*β* = − 0.13, *p* < 0.001), whereas BMI was positively associated with tensile stress (*β* = 0.21, *p* < 0.001). Age had no significant influence on tensile stress. Comparing the z-scores of tensile stress between the BMI groups, overweight girls and obese participants showed significantly higher tensile stress z-scores than normal weight children (girls: normal weight vs. overweight *p* = 0.031 and normal weight vs. obese *p* = 0.015; boys: normal weight vs. obese *p* < 0.001: Fig. S2 and Table S4 Online Resource).

## Discussion

This study provides age- and sex-dependent values for D, IDR, and tensile stress of the CCA for a sample of 642 healthy, non-obese children and adolescents. To the best of the authors’ knowledge, this is the first publication reporting D, IDR, and tensile stress values of the CCA in this population.

The findings for vascular parameters (IMT for the entire group 0.46 ± 0.03 mm; D girls 5.35 ± 0.42 mm, D boys 5.58 ± 0.47 mm) correspond well with those reported previously in the literature [40]. Nevertheless, conflicting results do exist due to methodological and population differences [22, 29, 40–43].

In multiple linear regression analysis, female sex was negatively associated with D and tensile stress but positively with IDR. In line with these findings, boys had higher D and tensile stress values than girls. In the literature, there were similar results for children [41, 42] and adolescents [44]. The sex difference in childhood for D is contrary to the findings for IMT, which seems not to differ between girls and boys in adolescence [31, 42], but only within adulthood [45]. Therefore, the authors of this study propose that the impact of sex on IDR and tensile stress may be predominantly based on interactions with arterial D. As the difference mainly occurs during the onset of puberty, hormonal influences on vascular tone and size could be a possible explanation for altered D values in boys and girls. Estrogen, progesterone, and testosterone receptors were detected in vascular cells in human arteries, which supports this hypothesis [46].

Consistent with the literature [42, 44], this study found significantly higher D z-scores for obese or overweight participants and a positive association of BMI with D and tensile stress. Even though both, IMT and D, are positively associated with a higher BMI [4], obese and overweight participants had a significantly lower IDR. Obesity is associated with increased blood flow and may lead to higher shear stress on the arterial wall [47]. This stress leads to an increased D through intracellular signaling processes [48]. In turn, an increased D may induce increased tensile stress with a subsequent IMT thickening [24]. Corresponding, a significantly higher tensile stress was found in obese and overweight girls and boys in this study population. Similar results were made by Chiesa et al. [29], who described an increased lumen, but a lower IMT with a reduced IDR and an increased tensile stress in obese young study participants. These findings indicate that risk factors for CVD, such as obesity, may be better explained by referring to IDR than IMT alone. Regarding the values of this healthy study population, a constant IDR over childhood can be assumed. In concordance, Chiesa et al. [29] suggested that increased fat-free mass is the predominant factor leading to a thickened IMT. In a growth-associated physiologic process, D increases as well—IDR, thereby, is kept at a constant level. Regarding the tensile stress in childhood, an inverse parabolic course of the graph can be observed in girls. The maximum tensile stress in 13–14-year-old girls matches the physiological growth in puberty. Furthermore, in boys, tensile stress seems to increase by the age of 15–17 years, which would also fit the physiological growth–associated process in boys. As growth is not finished, anthropometric alterations lead to altered blood flow and BP [49] and may be the reason for temporary altered tensile stress. A thickened IMT in childhood seems more likely to occur due to functional adaptation processes to altered vascular conditions, rather than being the expression of pathologic subclinical atherosclerosis. Further studies are needed to work on several issues: first, to confirm the increase of the IMT and D as functional processes, and, secondly, to identify the trigger factors inducing these changes.

The impact of age and BP on vascular parameters is unclear. This study did not find a significant influence of age on D, IDR, or tensile stress which is in line with the results of Sass et al. [42]. In contrast, a positive influence of age on D in childhood was described previously [40, 41]. Whether SBP or DBP is the predominant predictor for CVD remains unknown [50]. However, DBP seems to be the better parameter for evaluating the risk of CVD events at younger ages [51, 52]. CVD risk factors, such as sugar consumption [53], and IMT in young adolescents with insulin-dependent diabetes mellitus [53] are associated with increased DBP, but not SBP. Weberruss et al. [31] found a reduced acceleration of arterial stiffness and lower pulse pressure in children with higher DBP. The authors of the current study suggest a negative influence of SBP and a positive influence of DBP on D. Although only significant in girls, Sass et al. [42] found SBP to be a negative predictor of D. In contrast, hypertensive boys, but not girls, showed a significant higher D versus normotensive peers in a study by Litwin et al. [22] (5.2 ± 0.5 vs. 4.87 ± 0.5, *p* = 0.005). A positive influence of SBP on coronary IMT [31, 42] as well as on radial IMT [29] appears in the literature. These findings fit the current study results, revealing a positive influence of SBP on IDR. Further investigations are needed to elucidate the influencing factors on D and IDR.

The authors acknowledge several limitations of the present study. Presented values are based on a sample size of only 642 children. Separated by age group, the smallest age group cohort is 35 participants. Another methodologic limitation is the assessment of the vascular parameters on a single ultrasound machine, because these results may differ from measurements assessed with other ultrasound systems. The findings could only explain 9% of the variance of IDR with the parameters age, sex, BMI, SBP, and DBP. Especially for an adequate description of IDR, further influence factors on IMT and D must be taken into account. Higher IMT has been described in children and adolescents with the preload of familial hypercholesterolemia [6], increased concentrations of serum C-reactive protein [11], serum uric acid [54], plasma total homocysteine [55], increased cholesterol level [56], pubertal maturation [57], maternal obesity [58], parental smoking during pregnancy [59], severe intrauterine growth retardation [60], preterm birth [61, 62], and excess postnatal weight gain [63]. Therefore, these points should be investigated in future studies. Furthermore, participants with high BP values were not excluded from the analysis, although increased D and IMT were observed in children with hypertension [5]. To diagnose hypertension, at least three measurements of BP > 95th percentile must be assessed [64]. Because only one value was measured, this approach cannot exclude contributing factors such as “white-coat hypertension” [65]. The authors did not diagnose manifest, but suspected hypertension, in children with BP > 95th percentile.

## Conclusion

In conclusion, this study provides sex- and age-dependent values for D, IDR, and tensile stress of the CCA in 642 German children aged 8–17 years. These values may contribute to a more specific differentiation between the underlying processes of arterial wall alterations because both, structural and functional parameters are investigated.

## Electronic supplementary material

ESM 1 (DOCX 458 kb)

## Abbreviations

BMI: Body mass index
BP: Blood pressure
CCA: Common carotid artery
CVD: Cardiovascular disease
D: Vascular diameter
DBP: Diastolic blood pressure
IDR: Intima-media thickness/diameter ratio
IMT: Intima-media thickness
MAP: Mean arterial pressure
SBP: Systolic blood pressure
