# Correction to: Vascular diameter and intima-media thickness to diameter ratio values of the carotid artery in 642 healthy children

**DOI:** 10.1007/s00431-024-05865-0

**Published:** 2024-11-14

**Authors:** Luisa Semmler, Heidi Weberruß, Lisa Baumgartner, Raphael Pirzer, Renate Oberhoffer-Fritz

**Affiliations:** 1 Institute of Preventive Pediatrics, Technical University, Munich, Germany; 2 Department of Anaesthesiology and Operative Intensive Care, University Hospital, Augsburg, Germany

**Correction to: European Journal of Pediatrics (2020) 180:851-860**

10.1007/s00431-020-03785-3

This erratum serves to correct minor errors identified in our original publication. The numerical values of the parameter tensile stress were incorrectly stated throughout the manuscript due to an issue in the original R code. However, the conclusions drawn in the original paper are not altered. The following adaptions have been made and have already been updated in the online article:

Main text:

**Results:** The sentence “The tensile stress was 105.4 (95.2–116.4) kPa for the total study population, 103.3 (94.1–112.8) kPa for girls and 108.4 ± 16.2 kPa for boys.” should read “The tensile stress was 66.0 ± 8.8 kPa for the total study population, 64.9 ± 8.4 kPa for girls, and 67.2 ± 9.1 kPa for boys.” The same values were adapted in the last row of **Table 1** and the **Abstract**. Regarding the sex differences of tensile stress in the specific age groups, it should read “[…] 10.00–11.99 years and 12.00–13.99 years” instead of “[…] 10.00–11.99 years and 16.00–17.25 years”. Further, the sentence “[…] overweight and obese participants showed significantly higher tensile stress z-scores than normal weight children in both, girls and boys (girls: normal weight vs. overweight *p* = 0.022; boys normal weight vs. overweight *p* = 0.001; normal weight vs. obese for both *p* < 0.001” should read “[…] overweight girls and obese participants showed significantly higher tensile stress z-scores than normal weight children (girls: normal weight vs. overweight *p* = 0.031 and normal weight vs. obese *p* = 0.015; boys: normal weight vs. obese *p* < 0.001”.

In **Table 2** Model 3 (tensile stress) *β*, *β* st, *p* value and 95% CI of the parameters age, female sex and BMI were updated as following: for age from *β* = 1.89, *β* st = 0.08, *p* value = 0.059, and 95% CI = -0.02 to 1.22 to *β* = 0.04, *β* st = 0.01, *p* value = 0.809, and 95% CI = -0.30 to 0.39; for female sex from *β* = -3.93, *β* st = -0.15, *p* value < 0.001, and 95% CI = -7.22 to -2.41 to *β* = -2.38, *β* st = -0.13, *p* value < 0.001, and 95% CI = -3.73 to -1.03; and, for BMI from *β* = 6.68, *β* st = 0.29, *p* value < 0.001, and 95% CI = 1.22 to 2.23 to *β* = 0.68, *β* st = 0.21, *p* value < 0.001 and 95% CI = 0.40 to 0.97. R^2^ of the model should read 0.06 instead of 0.13.

Supplement:

In **Table S1** mean ± SD or median (IQR) of tensile stress for the total population, girls, and boys and the respective *p* values were updated as following: for age group 7.75–10.00 from 96.0 ± 15.3, 94.5 ± 15.6, 94.9 (88.9–105.5), *p* value 0.453 to 62.5 ± 8.4, 63.2 ± 8.5, 61.8 ± 8.4, *p* value 0.338, for age group 10.00–11.99 from 108.0 ± 15.9, 104.5 ± 15.3, 110.3 ± 15.8, *p* value 0.010 to 67.0 ± 9.2, 64.2 ± 7.8, 68.9 ± 9.5, *p* value < 0.001, for age group 12.00–13.99 from 108.5 (100.0–119.1), 105.9 (98.6–117.4), 110.4 (102.8–121.5), *p* value 0.064 to 67.9 ± 8.0, 66.3 ± 8.2, 69.8 ± 7.3, *p* value 0.006, for age group 14.00–15.99 from 106.1 (98.3–114.1), 106.2(98.8–113.8), 106.8 ± 15.2, *p* value 0.717 to 65.1 ± 8.6, 65.4 ± 8.9, 64.2 ± 7.6, *p* value 0.486, and age group 16.00–17.25 from 108.0 ± 18.6, 99.7 ± 17.3, 119.1 ± 14.0 *p* value 0.001 to 66.4 ± 9.5, 64.3 ± 9.2, 69.2 ± 9.4 *p* value 0.135.

In **Table S4** tensile stress z-scores were updated from 0.70 ± 1.08 to 0.58 ± 1.05 for overweight girls, from 0.97 (0.15–1.65) to 0.54 ± 1.22 for obese girls, from -0.07 ± 0.98 to -0.03 ± 1.00 for normal weight boys, from 0.64 ± 1.06 to 0.26 ± 0.96 for overweight boys, and from 0.89 ± 1.30 to 0.91 ± 0.97 for obese boys. *P* values were corrected from 0.022 to 0.031 for normal weight vs. overweight girls, from < 0.001 to 0.015 for normal weight vs. obese girls, from 0.001 to 0.399 for normal weight vs. overweight boys, and from 0.667 to 0.075 for overweight vs. obese boys. **Fig. S2** was updated, respectively.

Likewise, the numerical L, M, and S scores of tensile stress were corrected in **Table S7 for girls** and **Table S8** for boys. Corresponding smoothed fitted percentiles were updated in **Fig. S1**.

Further, the following typos have been identified and corrected:

Main text:

**Methods:** The z-score formula should read “z-score = [(x/M)^L]-1/LxS for L ≠ 0” instead of “z-score = (x/M)^L-1/LxS for L ≠ 0”.

**In Table 2** Model 2, the *p* value of age should read 0.204 instead of 0.187 and the *β* st of SBP 0.17 instead of 0.13. This was also adapted in the **Results** section.

**Results:** The* p* value referenced in the sentence “Comparing the z-scores of IDR between the BMI groups, in girls, overweight and obese participants showed significantly lower IDR z-scores versus normal weight children” should be “for all *p* < 0.01” instead of “for all *p* < 0.001”.

Supplement:

**Table S1**: In the age group 10.00–11.99, the diameter IQR of the total study population should read 5.18–5.83 instead of 5.17–5.83 and the diameter SD of boys should read 0.45 instead of 0.44. In the age group 14.00–15.99 the *p* value regarding sex differences in IDR should read 0.302 instead of 0.303.

**Table S4**: The *p* values comparing IDR z-scores should read 0.002 for normal weight vs. overweight girls instead of < 0.001 and 0.003 instead of < 0.001 for normal weight vs. obese girls.

These corrections do not alter the original findings of the article; rather, they ensure the accuracy of the results.

We sincerely apologize for any inconvenience this correction may have caused and appreciate your understanding.

Thank you for your attention to this matter.

The original article has been corrected.

