# Supplementary material for: Vascular diameter and intima-media thickness to diameter ratio values of the carotid artery in 642 healthy children
Source: Eur J Pediatr. 2020 Sep 18;180(3):851–60. doi: 10.1007/s00431-020-03785-3 (PMC7886737; doi:10.1007/s00431-020-03785-3)
Supplement: Supplementary file 1 — (DOCX 458 kb) [file 431_2020_3785_MOESM1_ESM.docx]

Vascular diameter and intima-media thickness to diameter ratio values of the carotid artery in 642 healthy children European Journal of Pediatrics

Luisa Semmler^[[1]](#footnote-2)^, Heidi Weberruß^1^, Lisa Baumgartner^1^, Raphael Pirzer^[[2]](#footnote-3)^, Renate Oberhoffer-Fritz^1^

Corresponding author: Luisa Semmler, E-Mail: [luisa.semmler@tum.de](mailto:luisa.semmler@tum.de).

**Supplemental Tables and Figures**

**Table S1** Differences between boys and girls in diameter, IDR and tensile stress, separated by age groups

**Table S2** Age-dependent L, M, S scores and values of vascular diameter in girls aged 8-17 years

**Table S3** Age-dependent L, M, S scores and values of vascular diameter in boys aged 8-17 years

**Table S4** Comparison of z-scores of diameter, IDR and tensile stress between BMI groups for girls and boys

**Table S5** Age dependent L, M, S scores and values of IDR in girls aged 8-17 years

**Table S6** Age dependent L, M, S scores and values of IDR in boys aged 8-17 years

**Table S7** Age dependent L, M, S scores and values of tensile stress in girls aged 8-17 years

**Table S8** Age dependent L, M, S scores and values of tensile stress in boys aged 8-17 years

**Fig. S1** Smoothed percentiles of tensile stress for girls and boys aged 8-17 years

**Fig. S2** Z-scores for tensile stress in normal weight, overweight, and obese girls and boys

**Table S1** Differences between boys and girls in diameter, IDR and tensile stress, separated by age groups

|  | total  n = 642 | girls  n = 344 | boys  n = 298 |  |
| --- | --- | --- | --- | --- |
| **Diameter [mm]** | | | | |
| age groups | mean±SD or median (IQR) | mean±SD or median (IQR) | mean±SD or median (IQR) | p-value |
| 7.75­-10.00  (n = 122) | 5.24±0.44 | 5.16±0.39 | 5.33±0.48 | 0.039 |
| 10.00-11.99  (n = 203) | 5.46 (5.18-5.83) | 5.25 (5.08-5.55) | 5.63±0.44 | <0.001 |
| 12.00-13.99  (n = 159) | 5.54±0.42 | 5.44±0.40 | 5.66±0.41 | 0.001 |
| 14.00-15.99  (n = 123) | 5.45±0.45 | 5.40±0.43 | 5.56±0.49 | 0.082 |
| 16.00-17.25  (n = 35) | 5.52±0.55 | 5.30±0.49 | 5.81±0.51 | 0.005 |
| total | 5.45±0.46 | 5.35±0.42 | 5.58±0.47 | <0.001 |
| **IDR** | | | | |
| age groups | mean±SD or median (IQR) | mean±SD or median (IQR) | mean±SD or median (IQR) | p-value |
| 7.75-10.00  (n = 122) | 0.087±0.008 | 0.087±0.008 | 0.087±0.009 | 0.919 |
| 10.00-11.99  (n = 203) | 0.085±0.009 | 0.088±0.009 | 0.083±0.008 | <0.001 |
| 12.00-13.99  (n = 159) | 0.085±0.008 | 0.086±0.008 | 0.081 (0.077-0.086) | 0.001 |
| 14.00-15.99  (n = 123) | 0.086 (0.079-0.094) | 0.088 (0.080-0.094) | 0.085±0.009 | 0.302 |
| 16.00-17.25  (n = 35) | 0.083 (0.079-0.090) | 0.086±0.009 | 0.083±0.010 | 0.372 |
| total | 0.085 (0.079-0.092) | 0.087±0.009 | 0.083 (0.078-0.090) | <0.001 |
| **Tensile stress [kPa]** | | | | |
| age groups | mean±SD or median (IQR) | mean±SD or median (IQR) | mean±SD or median (IQR) | p-value |
| 7.75-10.00  (n = 122) | 62.5±8.4 | 63.2±8.5 | 61.8±8.4 | 0.338 |
| 10.00-11.99  (n = 203) | 67.0±9.2 | 64.2±7.8 | 68.9±9.5 | <0.001 |
| 12.00-13.99  (n = 159) | 67.9±8.0 | 66.3±8.2 | 69.8±7.3 | 0.006 |
| 14.00-15.99  (n = 123) | 65.1±8.6 | 65.4±8.9 | 64.2±7.6 | 0.486 |
| 16.00-17.25  (n = 35) | 66.4±9.5 | 64.3±9.2 | 69.2±9.4 | 0.135 |
| total | 66.0±8.8 | 64.9±8.4 | 67.2±9.1 | <0.001 |
| p-values indicate sex differences in diameter, IDR and tensile stress, respectively. IDR = intima-media thickness:diameter ratio, IQR = interquartile range, n = number, SD = standard deviation | | | | |

**Table S2** Age dependent L, M, S scores and values of vascular diameter in girls aged 8-17 years

| Diameter [mm] | | | | | | | | | |
| --- | --- | --- | --- | --- | --- | --- | --- | --- | --- |
| Age [year] | P3 | P10 | P25 | P50 [M] | P75 | P90 | P97 | L | S |
| 8.0 | 4.42 | 4.60 | 4.80 | 5.06 | 5.35 | 5.65 | 6.00 | -1.56 | 0.08 |
| 8.5 | 4.47 | 4.65 | 4.85 | 5.10 | 5.38 | 5.67 | 6.00 | -1.45 | 0.08 |
| 9.0 | 4.52 | 4.70 | 4.89 | 5.14 | 5.41 | 5.69 | 6.00 | -1.34 | 0.07 |
| 9.5 | 4.57 | 4.74 | 4.94 | 5.18 | 5.45 | 5.72 | 6.02 | -1.23 | 0.07 |
| 10.0 | 4.61 | 4.79 | 4.98 | 5.22 | 5.49 | 5.75 | 6.04 | -1.12 | 0.07 |
| 10.5 | 4.64 | 4.82 | 5.02 | 5.26 | 5.53 | 5.79 | 6.07 | -1.01 | 0.07 |
| 11.0 | 4.67 | 4.85 | 5.05 | 5.30 | 5.56 | 5.83 | 6.11 | -0.91 | 0.07 |
| 11.5 | 4.69 | 4.88 | 5.09 | 5.33 | 5.60 | 5.86 | 6.15 | -0.80 | 0.07 |
| 12.0 | 4.71 | 4.90 | 5.11 | 5.36 | 5.63 | 5.90 | 6.18 | -0.69 | 0.07 |
| 12.5 | 4.73 | 4.93 | 5.14 | 5.39 | 5.67 | 5.93 | 6.21 | -0.58 | 0.07 |
| 13.0 | 4.75 | 4.95 | 5.16 | 5.42 | 5.70 | 5.96 | 6.24 | -0.47 | 0.07 |
| 13.5 | 4.75 | 4.95 | 5.17 | 5.44 | 5.71 | 5.98 | 6.27 | -0.36 | 0.07 |
| 14.0 | 4.73 | 4.94 | 5.17 | 5.44 | 5.72 | 5.99 | 6.27 | -0.25 | 0.07 |
| 14.5 | 4.70 | 4.91 | 5.15 | 5.42 | 5.71 | 5.98 | 6.27 | -0.15 | 0.08 |
| 15.0 | 4.64 | 4.87 | 5.11 | 5.39 | 5.69 | 5.97 | 6.26 | -0.04 | 0.08 |
| 15.5 | 4.58 | 4.82 | 5.07 | 5.36 | 5.67 | 5.96 | 6.25 | 0.07 | 0.08 |
| 16.0 | 4.52 | 4.77 | 5.03 | 5.33 | 5.64 | 5.94 | 6.25 | 0.18 | 0.09 |
| 16.5 | 4.46 | 4.71 | 4.98 | 5.30 | 5.63 | 5.93 | 6.25 | 0.29 | 0.09 |
| 17.0 | 4.39 | 4.66 | 4.94 | 5.27 | 5.61 | 5.93 | 6.25 | 0.40 | 0.09 |

**Table S3** Age dependent L, M, S scores and values of vascular diameter in boys aged 8-17 years

| Diameter [mm] | | | | | | | | | |
| --- | --- | --- | --- | --- | --- | --- | --- | --- | --- |
| Age [year] | P3 | P10 | P25 | P50 [M] | P75 | P90 | P97 | L | S |
| 8.0 | 4.37 | 4.65 | 4.92 | 5.22 | 5.52 | 5.77 | 6.02 | 1.44 | 0.08 |
| 8.5 | 4.44 | 4.72 | 4.99 | 5.29 | 5.59 | 5.85 | 6.10 | 1.35 | 0.08 |
| 9.0 | 4.51 | 4.78 | 5.06 | 5.36 | 5.66 | 5.92 | 6.18 | 1.25 | 0.08 |
| 9.5 | 4.58 | 4.85 | 5.13 | 5.43 | 5.72 | 5.99 | 6.25 | 1.16 | 0.08 |
| 10.0 | 4.65 | 4.92 | 5.19 | 5.49 | 5.79 | 6.06 | 6.32 | 1.07 | 0.08 |
| 10.5 | 4.71 | 4.98 | 5.25 | 5.55 | 5.85 | 6.12 | 6.39 | 0.98 | 0.08 |
| 11.0 | 4.77 | 5.03 | 5.30 | 5.60 | 5.90 | 6.17 | 6.44 | 0.89 | 0.08 |
| 11.5 | 4.81 | 5.07 | 5.34 | 5.63 | 5.94 | 6.21 | 6.48 | 0.80 | 0.08 |
| 12.0 | 4.83 | 5.09 | 5.35 | 5.65 | 5.95 | 6.23 | 6.50 | 0.71 | 0.08 |
| 12.5 | 4.84 | 5.10 | 5.36 | 5.65 | 5.96 | 6.23 | 6.51 | 0.62 | 0.08 |
| 13.0 | 4.84 | 5.09 | 5.35 | 5.65 | 5.95 | 6.23 | 6.52 | 0.53 | 0.08 |
| 13.5 | 4.84 | 5.09 | 5.35 | 5.64 | 5.95 | 6.23 | 6.52 | 0.44 | 0.08 |
| 14.0 | 4.83 | 5.08 | 5.34 | 5.64 | 5.95 | 6.24 | 6.53 | 0.34 | 0.08 |
| 14.5 | 4.83 | 5.08 | 5.34 | 5.64 | 5.95 | 6.24 | 6.54 | 0.25 | 0.08 |
| 15.0 | 4.83 | 5.08 | 5.34 | 5.65 | 5.96 | 6.26 | 6.57 | 0.16 | 0.08 |
| 15.5 | 4.84 | 5.09 | 5.35 | 5.66 | 5.98 | 6.29 | 6.60 | 0.07 | 0.08 |
| 16.0 | 4.86 | 5.11 | 5.37 | 5.68 | 6.01 | 6.32 | 6.64 | -0.02 | 0.08 |
| 16.5 | 4.88 | 5.13 | 5.39 | 5.71 | 6.04 | 6.36 | 6.69 | -0.11 | 0.08 |
| 17.0 | 4.90 | 5.15 | 5.42 | 5.73 | 6.08 | 6.40 | 6.75 | -0.20 | 0.09 |

**Table S4** Comparison of z-scores of diameter IDR and tensile stress between BMI groups for girls and boys

| z-scores (mean±SD or median (IQR)) | | | | p-values | | |
| --- | --- | --- | --- | --- | --- | --- |
|  | normal weight | overweight | obese | normal weight vs. overweight | normal weight vs. obese | overweight vs. obese |
| **girls** | | | | | | |
| D | -0.07±0.97 | 1.24±1.09 | 1.29 (0.17-2.04) | <0.001 | <0.001 | 1 |
| IDR | 0.05±0.97 | -0.75±1.19 | -0.62±1.12 | 0.002 | 0.003 | 1 |
| tensile stress | -0.04±0.99 | 0.58±1.05 | 0.54±1.22 | 0.031 | 0.015 | 1 |
| **boys** | | | | | | |
| D | -0.08±0.98 | 0.69±0.97 | 0.97±0.96 | <0.001 | <0.001 | 1 |
| IDR | 0.03±1.01 | -0.25±0.86 | -0.78±1.08 | 0.428 | 0.002 | 0.227 |
| tensile stress | -0.03±1.00 | 0.26±0.96 | 0.91±0.97 | 0.399 | <0.001 | 0.075 |
| Z-scores of diameter, IDR and tensile stress were compared between normal weight, overweight and obese subjects. p-values indicate differences between the groups. D = diameter, IDR = intima-media thickness:diameter ratio, IQR = interquartile range, SD = standard deviation | | | | | | |

**Table S5** Age dependent L, M, S scores and values of IDR in girls aged 8-17 years

| IDR | | | | | | | | | |
| --- | --- | --- | --- | --- | --- | --- | --- | --- | --- |
| Age [year] | P3 | P10 | P25 | P50 [M] | P75 | P90 | P97 | L | S |
| 8.0 | 0.072 | 0.077 | 0.082 | 0.087 | 0.093 | 0.097 | 0.102 | 1.500 | 0.091 |
| 8.5 | 0.072 | 0.077 | 0.082 | 0.087 | 0.093 | 0.097 | 0.102 | 1.361 | 0.092 |
| 9.0 | 0.072 | 0.077 | 0.082 | 0.087 | 0.093 | 0.098 | 0.102 | 1.230 | 0.093 |
| 9.5 | 0.071 | 0.077 | 0.082 | 0.087 | 0.093 | 0.098 | 0.103 | 1.113 | 0.095 |
| 10.0 | 0.071 | 0.076 | 0.081 | 0.087 | 0.093 | 0.098 | 0.103 | 1.014 | 0.096 |
| 10.5 | 0.071 | 0.076 | 0.081 | 0.087 | 0.093 | 0.098 | 0.103 | 0.933 | 0.097 |
| 11.0 | 0.071 | 0.076 | 0.081 | 0.087 | 0.093 | 0.098 | 0.103 | 0.868 | 0.099 |
| 11.5 | 0.071 | 0.076 | 0.081 | 0.087 | 0.093 | 0.098 | 0.104 | 0.812 | 0.100 |
| 12.0 | 0.071 | 0.076 | 0.081 | 0.087 | 0.093 | 0.098 | 0.104 | 0.754 | 0.101 |
| 12.5 | 0.071 | 0.076 | 0.081 | 0.087 | 0.093 | 0.098 | 0.104 | 0.680 | 0.103 |
| 13.0 | 0.070 | 0.075 | 0.081 | 0.087 | 0.093 | 0.099 | 0.104 | 0.573 | 0.104 |
| 13.5 | 0.070 | 0.075 | 0.081 | 0.087 | 0.093 | 0.099 | 0.105 | 0.408 | 0.106 |
| 14.0 | 0.070 | 0.075 | 0.080 | 0.087 | 0.093 | 0.099 | 0.105 | 0.171 | 0.107 |
| 14.5 | 0.071 | 0.075 | 0.080 | 0.086 | 0.093 | 0.099 | 0.106 | -0.139 | 0.109 |
| 15.0 | 0.071 | 0.075 | 0.080 | 0.086 | 0.093 | 0.100 | 0.107 | -0.506 | 0.110 |
| 15.5 | 0.071 | 0.075 | 0.080 | 0.086 | 0.093 | 0.101 | 0.109 | -0.911 | 0.112 |
| 16.0 | 0.071 | 0.076 | 0.080 | 0.086 | 0.093 | 0.101 | 0.111 | -1.334 | 0.113 |
| 16.5 | 0.072 | 0.076 | 0.080 | 0.086 | 0.094 | 0.102 | 0.113 | -1.763 | 0.115 |
| 17.0 | 0.072 | 0.076 | 0.080 | 0.086 | 0.094 | 0.103 | 0.116 | -2.193 | 0.116 |

**Table S6** Age dependent L, M, S scores and values of IDR in boys aged 8-17 years

| IDR | | | | | | | | | |
| --- | --- | --- | --- | --- | --- | --- | --- | --- | --- |
| Age [year] | P3 | P10 | P25 | P50 [M] | P75 | P90 | P97 | L | S |
| 8.0 | 0.074 | 0.078 | 0.083 | 0.089 | 0.094 | 0.100 | 0.106 | -0.169 | 0.095 |
| 8.5 | 0.073 | 0.077 | 0.082 | 0.087 | 0.093 | 0.099 | 0.105 | -0.213 | 0.096 |
| 9.0 | 0.072 | 0.076 | 0.081 | 0.086 | 0.092 | 0.098 | 0.104 | -0.257 | 0.096 |
| 9.5 | 0.071 | 0.075 | 0.080 | 0.085 | 0.091 | 0.096 | 0.102 | -0.301 | 0.097 |
| 10.0 | 0.070 | 0.074 | 0.079 | 0.084 | 0.090 | 0.095 | 0.101 | -0.345 | 0.097 |
| 10.5 | 0.070 | 0.074 | 0.078 | 0.083 | 0.089 | 0.095 | 0.101 | -0.390 | 0.098 |
| 11.0 | 0.069 | 0.073 | 0.077 | 0.082 | 0.088 | 0.094 | 0.100 | -0.434 | 0.098 |
| 11.5 | 0.069 | 0.073 | 0.077 | 0.082 | 0.088 | 0.093 | 0.100 | -0.478 | 0.099 |
| 12.0 | 0.068 | 0.072 | 0.077 | 0.082 | 0.088 | 0.093 | 0.099 | -0.522 | 0.099 |
| 12.5 | 0.068 | 0.072 | 0.077 | 0.082 | 0.088 | 0.093 | 0.100 | -0.566 | 0.100 |
| 13.0 | 0.069 | 0.072 | 0.077 | 0.082 | 0.088 | 0.094 | 0.100 | -0.610 | 0.100 |
| 13.5 | 0.069 | 0.073 | 0.077 | 0.082 | 0.088 | 0.094 | 0.101 | -0.654 | 0.101 |
| 14.0 | 0.069 | 0.073 | 0.077 | 0.082 | 0.088 | 0.094 | 0.101 | -0.698 | 0.101 |
| 14.5 | 0.069 | 0.073 | 0.077 | 0.083 | 0.089 | 0.095 | 0.102 | -0.743 | 0.102 |
| 15.0 | 0.069 | 0.073 | 0.078 | 0.083 | 0.089 | 0.095 | 0.102 | -0.787 | 0.102 |
| 15.5 | 0.070 | 0.073 | 0.078 | 0.083 | 0.089 | 0.096 | 0.103 | -0.831 | 0.103 |
| 16.0 | 0.070 | 0.073 | 0.078 | 0.083 | 0.089 | 0.096 | 0.103 | -0.875 | 0.103 |
| 16.5 | 0.070 | 0.073 | 0.078 | 0.083 | 0.089 | 0.096 | 0.103 | -0.919 | 0.104 |
| 17.0 | 0.069 | 0.073 | 0.078 | 0.083 | 0.089 | 0.096 | 0.103 | -0.963 | 0.104 |

**Table S7** Age dependent L, M, S scores and values of tensile stress in girls aged 8-17 years

| Tensile stress [kPa] | | | | | | | | | |
| --- | --- | --- | --- | --- | --- | --- | --- | --- | --- |
| Age [year] | P3 | P10 | P25 | P50 [M] | P75 | P90 | P97 | L | S |
| 8.0 | 47.50 | 52.28 | 57.28 | 62.98 | 68.86 | 74.28 | 79.75 | 0.69 | 0.14 |
| 8.5 | 48.05 | 52.73 | 57.61 | 63.17 | 68.88 | 74.14 | 79.45 | 0.70 | 0.13 |
| 9.0 | 48.57 | 53.17 | 57.93 | 63.36 | 68.93 | 74.05 | 79.20 | 0.72 | 0.13 |
| 9.5 | 49.05 | 53.57 | 58.25 | 63.58 | 69.03 | 74.03 | 79.06 | 0.73 | 0.13 |
| 10.0 | 49.47 | 53.94 | 58.56 | 63.81 | 69.18 | 74.10 | 79.05 | 0.74 | 0.12 |
| 10.5 | 49.83 | 54.27 | 58.87 | 64.08 | 69.39 | 74.26 | 79.15 | 0.75 | 0.12 |
| 11.0 | 50.12 | 54.56 | 59.15 | 64.35 | 69.65 | 74.51 | 79.37 | 0.77 | 0.12 |
| 11.5 | 50.33 | 54.80 | 59.42 | 64.64 | 69.96 | 74.82 | 79.69 | 0.78 | 0.12 |
| 12.0 | 50.47 | 54.99 | 59.65 | 64.92 | 70.28 | 75.18 | 80.08 | 0.79 | 0.12 |
| 12.5 | 50.54 | 55.12 | 59.85 | 65.18 | 70.61 | 75.56 | 80.52 | 0.80 | 0.12 |
| 13.0 | 50.50 | 55.17 | 59.98 | 65.41 | 70.92 | 75.95 | 80.98 | 0.82 | 0.12 |
| 13.5 | 50.34 | 55.12 | 60.03 | 65.57 | 71.20 | 76.32 | 81.44 | 0.83 | 0.13 |
| 14.0 | 50.06 | 54.96 | 59.99 | 65.66 | 71.41 | 76.64 | 81.87 | 0.84 | 0.13 |
| 14.5 | 49.66 | 54.69 | 59.85 | 65.66 | 71.55 | 76.91 | 82.26 | 0.85 | 0.13 |
| 15.0 | 49.16 | 54.33 | 59.64 | 65.61 | 71.65 | 77.15 | 82.63 | 0.87 | 0.14 |
| 15.5 | 48.61 | 53.93 | 59.38 | 65.52 | 71.72 | 77.36 | 82.98 | 0.88 | 0.14 |
| 16.0 | 48.03 | 53.50 | 59.11 | 65.41 | 71.78 | 77.57 | 83.32 | 0.89 | 0.14 |
| 16.5 | 47.44 | 53.07 | 58.84 | 65.31 | 71.85 | 77.78 | 83.68 | 0.90 | 0.15 |
| 17.0 | 46.82 | 52.63 | 58.56 | 65.21 | 71.92 | 78.01 | 84.06 | 0.91 | 0.15 |

**Table S8** Age dependent L, M, S scores and values of tensile stress in boys aged 8-17 years

| Tensile stress [kPa] | | | | | | | | | |
| --- | --- | --- | --- | --- | --- | --- | --- | --- | --- |
| Age [year] | P3 | P10 | P25 | P50 [M] | P75 | P90 | P97 | L | S |
| 8.0 | 47.00 | 50.38 | 54.24 | 59.14 | 64.84 | 70.80 | 77.63 | -0.67 | 0.13 |
| 8.5 | 47.76 | 51.26 | 55.24 | 60.22 | 65.93 | 71.81 | 78.43 | -0.52 | 0.13 |
| 9.0 | 48.50 | 52.14 | 56.22 | 61.29 | 67.01 | 72.81 | 79.23 | -0.38 | 0.13 |
| 9.5 | 49.42 | 53.22 | 57.43 | 62.60 | 68.35 | 74.10 | 80.36 | -0.23 | 0.13 |
| 10.0 | 50.63 | 54.60 | 58.97 | 64.27 | 70.09 | 75.82 | 81.97 | -0.08 | 0.13 |
| 10.5 | 51.92 | 56.09 | 60.63 | 66.07 | 71.97 | 77.69 | 83.76 | 0.07 | 0.13 |
| 11.0 | 53.27 | 57.64 | 62.36 | 67.95 | 73.93 | 79.65 | 85.64 | 0.22 | 0.13 |
| 11.5 | 54.42 | 59.00 | 63.88 | 69.60 | 75.63 | 81.34 | 87.23 | 0.36 | 0.13 |
| 12.0 | 54.81 | 59.53 | 64.52 | 70.28 | 76.29 | 81.90 | 87.63 | 0.51 | 0.12 |
| 12.5 | 54.42 | 59.23 | 64.24 | 69.97 | 75.87 | 81.31 | 86.81 | 0.66 | 0.12 |
| 13.0 | 53.68 | 58.54 | 63.56 | 69.22 | 74.97 | 80.22 | 85.47 | 0.81 | 0.12 |
| 13.5 | 52.70 | 57.61 | 62.60 | 68.17 | 73.75 | 78.80 | 83.79 | 0.96 | 0.12 |
| 14.0 | 51.52 | 56.45 | 61.39 | 66.84 | 72.25 | 77.07 | 81.81 | 1.10 | 0.12 |
| 14.5 | 50.45 | 55.41 | 60.32 | 65.66 | 70.90 | 75.53 | 80.03 | 1.25 | 0.12 |
| 15.0 | 49.86 | 54.90 | 59.83 | 65.12 | 70.24 | 74.73 | 79.05 | 1.40 | 0.12 |
| 15.5 | 50.03 | 55.24 | 60.26 | 65.57 | 70.66 | 75.08 | 79.30 | 1.55 | 0.12 |
| 16.0 | 50.97 | 56.44 | 61.63 | 67.06 | 72.20 | 76.61 | 80.80 | 1.70 | 0.12 |
| 16.5 | 52.47 | 58.28 | 63.70 | 69.30 | 74.54 | 79.01 | 83.21 | 1.84 | 0.12 |
| 17.0 | 54.26 | 60.46 | 66.15 | 71.96 | 77.33 | 81.87 | 86.11 | 1.99 | 0.11 |

**Fig. S1** Smoothed percentiles of tensile stress for girls and boys aged 8-17 years


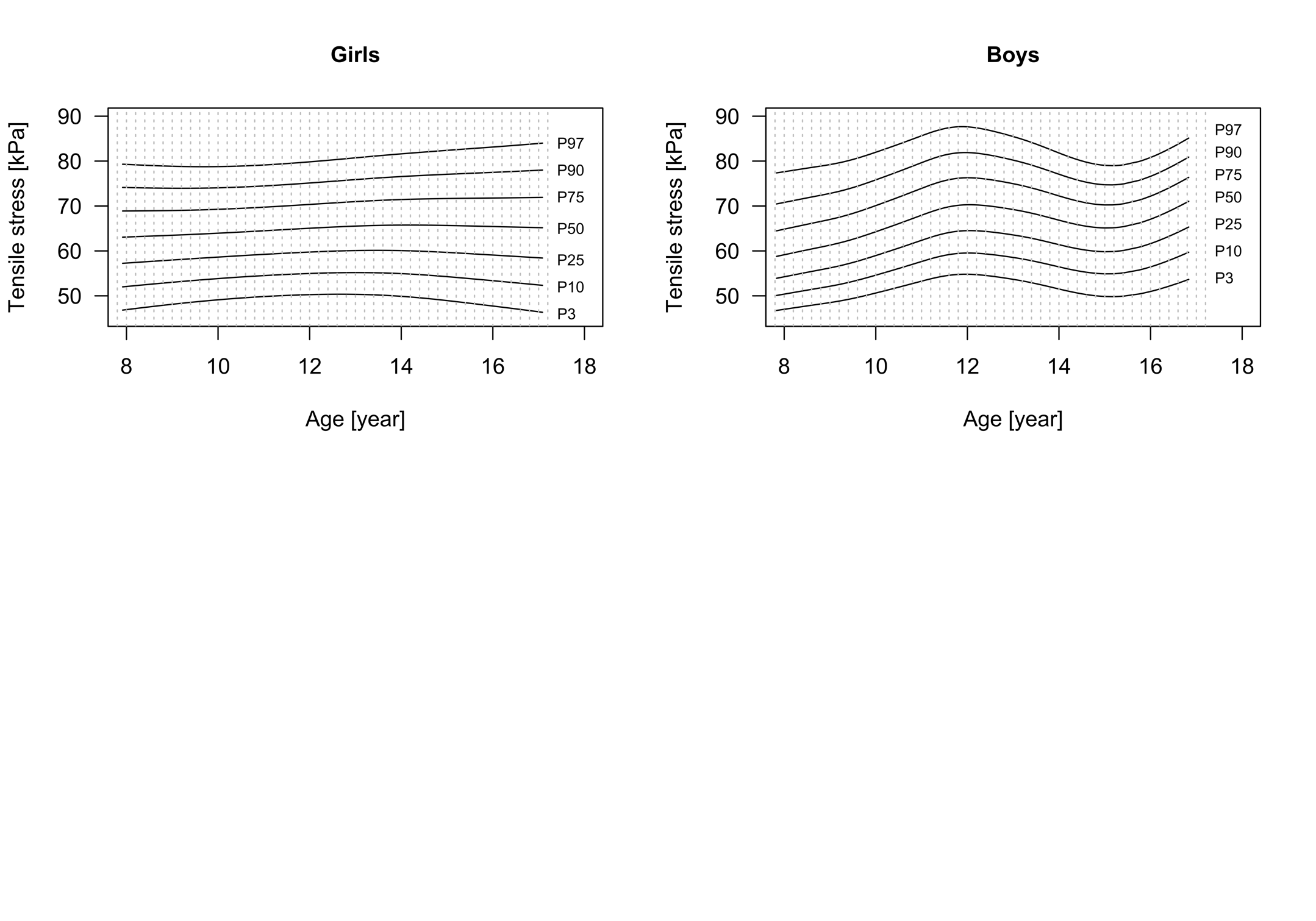


**Fig. S2** Z-scores for tensile stress in normal weight, overweight, and obese girls and boys


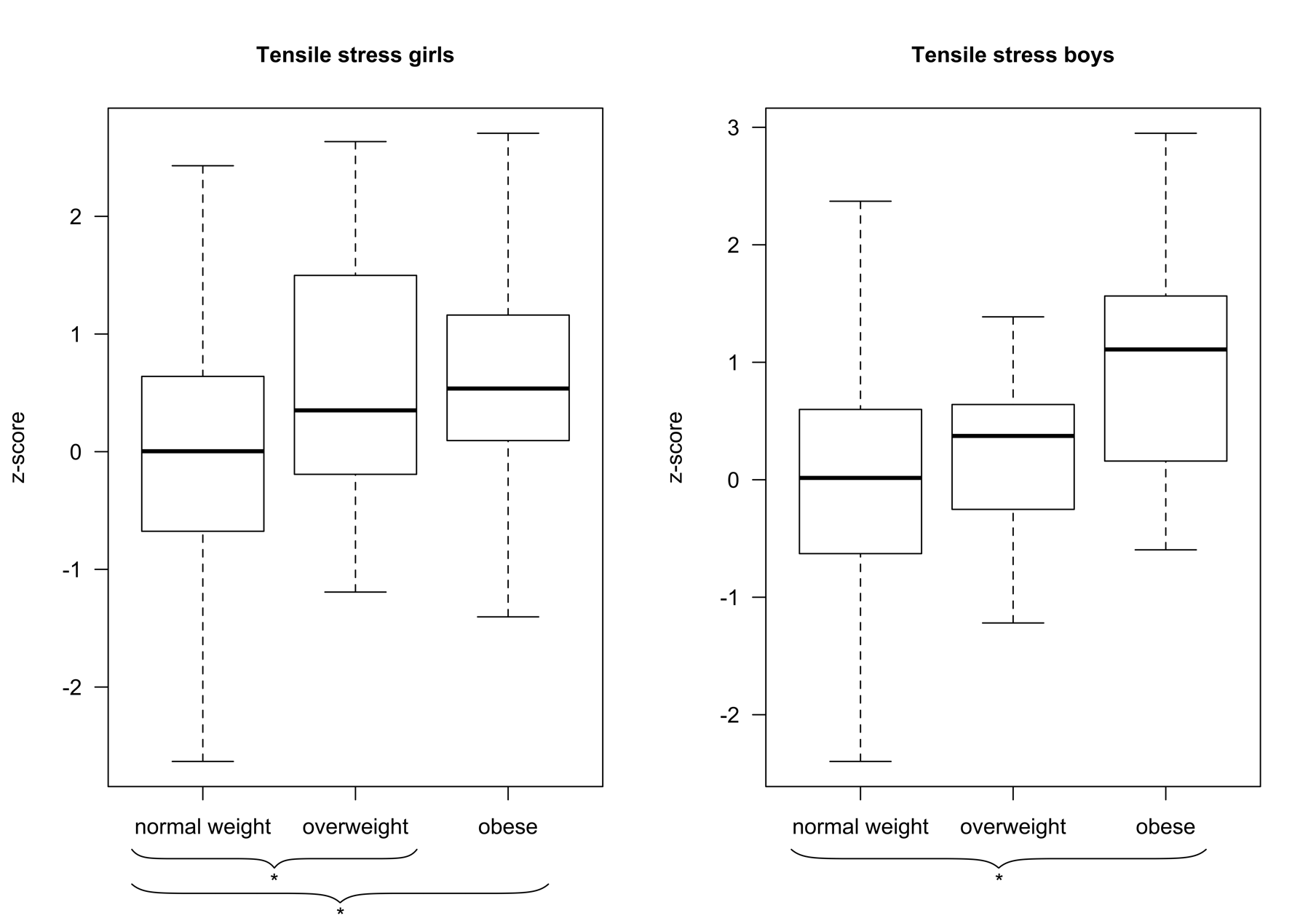


* p<0.05

1. Institute of Preventive Pediatrics, Technical University, Munich, Germany [↑](#footnote-ref-2)
2. Department of Anaesthesiology and Operative Intensive Care, University Hospital, Augsburg, Germany [↑](#footnote-ref-3)
